# Adapting home-based records for maternal and child health to users' capacities

**DOI:** 10.2471/BLT.18.216119

**Published:** 2019-02-14

**Authors:** Keiko Osaki, Hirotsugu Aiga

**Affiliations:** a Japan International Cooperation Agency, Nibancho Center Building, 5–25, Niban-cho, Chiyoda-ku, Tokyo 102-8012, Japan.

## Abstract

Home-based records have been used in both low- and high-income countries to improve maternal and child health. Traditionally, these were mostly stand-alone records that supported a single maternal and child health-related programme, such as the child vaccination card or growth chart. Recently, an increasing number of countries are using integrated home-based records to support all or part of maternal and child health-related programmes, as in the maternal and child health handbook. Policy-makers’ expectations of home-based records are often unrealistic and important functions of the records remain underused, leading to loss of confidence in the process, and to wasted resources and opportunities for care. We need to examine the gaps between the functions of the records and the extent to which users of records (pregnant women, mothers, caregivers and health-care workers) are knowledgeable and skilful enough to make those expected functions happen. Three key functions, with increasing levels of complexity, may be planned in home-based records: (i) data recording and storage; (ii) behaviour change communication, and (iii) monitoring and referral. We define a function–capacity conceptual framework for home-based records showing how increasing number and complexity of functions in a home-based record requires greater capacity among its users. The type and functions of an optimal home-based record should be strategically selected in accordance not only with demands of the health system, but also the capacities of the record users.

## Résumé

Les fiches de santé tenues à domicile sont aussi bien utilisées dans des pays à revenu faible que dans des pays à revenu élevé pour améliorer la santé de la mère et de l'enfant. Habituellement, les fiches utilisées étaient essentiellement des documents indépendants, associés à un programme spécifique de santé de la mère et de l'enfant, tels que les carnets de vaccination ou les courbes de croissance des enfants. Depuis quelque temps, un nombre croissant de pays opte pour des fiches plus complètes, couvrant tout ou partie des programmes de santé de la mère et de l'enfant, comme les manuels de santé de la mère et de l'enfant. Les attentes des décideurs politiques autour des fiches de santé tenues à domicile sont souvent trop ambitieuses, et des fonctions importantes des fiches sont sous-employées, ce qui rend le processus moins fiable, gâche des ressources et fait perdre des opportunités de soins. Il est nécessaire d’examiner l’écart entre les fonctions des fiches et le degré de connaissance et de compétence de leurs utilisateurs (femmes enceintes, mères, aidants, professionnels de santé) pour véritablement tirer parti de toutes les fonctions prévues. Trois principales fonctions, énoncées dans l'ordre croissant de leur degré de complexité, doivent être prévues dans les fiches de santé tenues à domicile: (i) enregistrement et stockage des données; (ii) communication des changements des comportements et (iii) suivi et recommandations pour le parcours de soins. Nous avons défini un cadre conceptuel Fonction-Capacité pour les fiches de santé tenues à domicile, qui montre comment des fonctions plus nombreuses et plus complexes nécessitent de plus grandes capacités chez les utilisateurs. Le type et les fonctions d'une fiche de santé optimale devraient être choisis stratégiquement en fonction, non seulement des exigences des systèmes de santé, mais aussi des capacités des utilisateurs des fiches.

## Resumen

Los registros domiciliarios se han utilizado tanto en países de bajos ingresos como en los de altos ingresos para mejorar la salud materno-infantil. Tradicionalmente, se trataba en su mayoría de registros independientes que apoyaban un único programa relacionado con la salud materno-infantil, como la tarjeta de vacunación infantil o el gráfico de crecimiento. Hace poco, un número cada vez mayor de países ha empezado a utilizar registros integrados domiciliarios para apoyar todos o una parte de los programas relacionados con la salud materno-infantil, como en el manual de salud materno-infantil. Las expectativas de los responsables de la formulación de políticas con respecto a los registros domiciliarios son a menudo demasiado ambiciosas y muchas funciones importantes de los registros no se aprovechan, lo que conduce a una pérdida de confianza en el proceso y a un despilfarro de recursos y oportunidades de atención. Es necesario examinar las brechas entre las funciones de los registros y la medida en que los usuarios de los mismos (mujeres embarazadas, madres, cuidadores y trabajadores de la salud) tienen el conocimiento y la habilidad suficientes para que esas funciones se cumplan. Se pueden planificar tres funciones clave, con niveles crecientes de complejidad, en los registros domiciliarios: (i) registro y almacenamiento de datos, (ii) comunicación de cambios de comportamiento, y (iii) seguimiento y remisión. Se ha definido un marco conceptual de función y capacidad para los registros domiciliarios que muestra cómo un número y una complejidad crecientes de las funciones en un registro domiciliario requiere una mayor capacidad de sus usuarios. El tipo y las funciones de un registro domiciliario óptimo deben seleccionarse estratégicamente de acuerdo no solo con las demandas del sistema de salud, sino también con las capacidades de los usuarios del registro.

## ملخص

تم استخدام السجلات المنزلية في كل من البلدان ذات الدخل المنخفض والدخل المرتفع لتحسين صحة الأم والطفل. وعلى نحو تقليدي، كانت هذه في الغالب سجلات قائمة بذاتها تدعم برنامجًا واحدًا متعلق بصحة الأم والطفل، مثل بطاقة تطعيم الطفل أو مخطط النمو الخاص به. وحديثاً، يستخدم عدد متزايد من البلدان السجلات المنزلية المتكاملة لدعم كل أو جزء من البرامج المتعلقة بصحة الأم والطفل، كما في كتيب صحة الأم والطفل. وغالباً ما تكون توقعات صناع القرار من السجلات المنزلية مفرطة في الطموح، ولا تزال الوظائف الهامة للسجلات غير مستغلة، مما يؤدي إلى فقدان الثقة في العملية، وإهدار الموارد وفرص الرعاية. نحن بحاجة إلى دراسة الفجوات بين وظائف السجلات ومدى دراية ومهارة مستخدمي السجلات (النساء الحوامل والأمهات ومقدمي الرعاية والعاملين في الرعاية الصحية) بقدر كاف مما يسهم في تفعيل هذه الوظائف. يمكن التخطيط لثلاث وظائف رئيسية في السجلات المنزلية، ذات مستويات متزايدة في التعقيد: (1) تسجيل البيانات وتخزينها؛ و(2) تواصل تغيير السلوك، و(3) والمراقبة والإحالة. نحن نحدد إطار عمل مفاهيمي للقدرة الوظيفية للسجلات المنزلية، مما يوضح أن التعدد المتزايد والتعقيد في وظائف السجلات المنزلية، يتطلب قدرة أكبر بين مستخدميها. يجب اختيار نوع وظائف السجل المنزلي المثالي ووظائفه بشكل استراتيجي بما يتوافق، ليس فقط مع مطالب النظام الصحي، ولكن أيضا مع قدرات مستخدمي السجلات.

## 摘要

低收入和高收入国家均使用基于家庭的记录来改善孕产妇和儿童的健康。传统上,这些独立记录大多是支持与孕产妇和儿童健康相关的单一记录,例如儿童疫苗接种卡或儿童生长表。最近,越来越多的国家正在使用基于家庭的综合记录来支持所有或部分与孕产妇和儿童健康相关的计划,例如母婴健康手册。政策制定者对基于家庭的记录往往期望过高,而不能充分运用其重要功能,导致对该进程失去信心,并浪费护理资源和机会。我们需要检查记录的功能与记录使用者(孕产妇、母亲、看护人和医疗保健工作者)的知识渊博和技术娴熟程度之间的差距,以实现预期功能。随着复杂性的增加,可以在基于家庭的记录中规划三项关键功能:(i)数据记录和存储;(ii)行为改变沟通,以及(iii)监测和转诊。我们为基于家庭的记录定义了一个功能——能力概念框架,显示了基于家庭的记录中,日益增加的功能数量和复杂性程度如何要求其使用者具备更强大的能力。基于家庭的最佳记录类型和功能不仅应根据卫生系统的需求,而且应综合记录使用者所具备的能力进行战略性选择。

## Резюме

Хранимые на дому записи о здоровье используются в странах как с высоким, так и с низким уровнем дохода для совершенствования системы охраны здоровья матери и ребенка. Традиционно это были в большинстве своем отдельные записи, которые способствовали поддержке единой программы охраны здоровья матери и ребенка, например карта вакцинации или карта физического развития. С недавних пор все большее количество стран используют хранимые на дому записи в качестве элемента полной или частичной поддержки программ по охране здоровья матери и ребенка, например руководство по охране здоровья матери и ребенка. Часто ожидания тех, кто принимает стратегические решения, по поводу таких хранимых на дому записей оказываются чрезмерно завышенными, а важные функции записей используются не в полной мере, что приводит к утрате доверия к процессу, напрасной трате ресурсов и возможностей получения медицинской помощи. Необходимо изучить расхождения между целями записей и тем, в какой мере пользователи таких записей (беременные женщины, матери, лица, осуществляющие уход, и работники здравоохранения) обладают навыками и знаниями, необходимыми для реализации ожидаемых целей. Можно запланировать три основных мероприятия (с возрастающим уровнем сложности) по ведению хранимых на дому записей о здоровье: (I) ведение записей и их хранение; (ii) просветительская работа в целях изменения моделей поведения; (iii) мониторинг и направление к специалистам. Авторы определили концептуальную модель целей и способностей применительно к хранимым на дому записям о здоровье, которая демонстрирует, что увеличение количества целей хранимых на дому записей и повышение их сложности требует совершенствования способностей пользователей. Тип и цели оптимальной системы хранимых на дому записей о здоровье должны определяться стратегически с учетом не только потребностей системы здравоохранения, но и умений и навыков пользователей записей.

## Introduction

In September 2018, the World Health Organization (WHO) launched its *Recommendations on home-based records for maternal, newborn and child health*.^1^ The guidelines reconfirmed the effectiveness of home-based records in increasing the use of maternal and child health services. This initiative by WHO provides the global health community with an opportunity to revisit what these records are and how they should be designed and implemented.

A home-based record is a health document used to record the history of health services received by an individual. The record is kept in the household, in paper or electronic format, by the individual or his or her caregivers. The record is intended to be integrated into the routine health information system and to complement records maintained by health facilities. Home-based records have been used in over 163 countries, notably for the improvement of maternal and child health.^1^ Traditionally, maternal and child health programmes have developed their own programme-specific home-based records and implemented these in parallel as an essential part of their vertical service-delivery systems. Examples include child vaccination cards for child immunization programmes,^2^^,^^3^ growth charts for child nutrition programmes^4^ and maternal health cards for reproductive and maternal health programmes.^5^ In some countries, the child vaccination card and growth chart are integrated into a child-targeted home-based record, often called the child health card or handbook.^1^ In many countries, the maternal health card continues to be a stand-alone record covering pregnancy, childbirth and the postpartum stages, independent from child records. We have observed that this difference in target groups has prompted policy debates on whether home-based records should be developed and distributed per child or per mother. Moreover, even if the consensus is for full integration of all the maternal and child records into a single record, poor coordination across departments within a health ministry can make it difficult to enforce such integration due to the nature of vertical programmes. Most countries therefore prefer to implement a series of programme-specific, stand-alone home-based records for maternal and child health services.

In efforts to address the millennium development goals, there has been an increasing interest in integrated records that cover the entire spectrum of pregnancy, childbirth, postpartum and newborn care, through to childhood and, sometimes, adolescence. As of 2016, at least 25 countries used fully integrated home-based records, often called the maternal and child health handbook.^6^ Key advantages of full integration include greater assurance of a continuum of maternal and child health care^7^^–^^10^ and major cost savings in the operation of the records.^11^ Fully integrated home-based records have been attracting more attention from health ministries^12^^,^^13^ and professional organizations^14^^,^^15^ as an effective tool for promoting a life-course approach to health care. Such an approach is conducive to achieving sustainable development goal 3 to “ensure healthy lives and promote well-being for all at all ages.”^16^

Despite the important role to be played by home-based records, there are a limited number of studies of the management of home-based records and most are focused on child vaccination cards. Nevertheless, some challenges to setting up effective and efficient home-based records have been identified. First, ensuring efficient, sustainable supplies of records is needed. Studies have highlighted the vulnerability of financing for home-based records;^17^ poor stock management of records at national, provincial, district and facility levels;^18^ and fragmented and parallel operations of multiple records.^19^ Second, the availability of and access to home-based records differs between and within countries.^2^ Sub-national analyses of the management of home-based records find that the availability of and access to these records differs between low- and high-income groups,^20^ between rural and urban areas,^21^ and between less and more educated mothers.^21^ Third, studies have reported misuse or poor use of home-based records by both service users (such as pregnant women, mothers and other caregivers) and service providers (such as health-care workers). For instance, mothers who are indifferent to home-based records are likely to damage, misplace or lose the records.^2^ Some health-care workers are careless about recording or reluctant to record the results of health check-ups, diagnosis and treatment in the home-based record.^9^

The question why misuse or inadequate use of home-based records takes place has not yet been clearly answered. We hypothesize that poor use of home-based records might be attributable to a mismatch between their expected functions and the reality of users’ capacities to manage the records. One study on the diverse functions of home-based records discussed three possible functions of child vaccination cards: (i) to increase mothers’ knowledge about and demand for health-care services; (ii) to facilitate communication between mothers and health-care workers; and (iii) to reduce missed opportunities for health-care services.^22^ The lack of studies on the relationship between the functions of home-based records and users’ capacities makes it difficult to devise strategies for optimal use of such records. We propose that the users of home-based records need a specific set of capacities to enable the expected functions of the records to take place. WHO’s recent guidelines, although a welcome initiative, recommend appropriate use of home-based records without also emphasizing the relationship between expected functions and users’ capacities.^1^

In this paper, we summarize the expected functions of home-based records and propose a function–capacity conceptual framework for home-based records.

## Functions of home-based records

We reviewed earlier studies on the operation of different types of home-based records, to explore differences and similarities in their expected functions. We conducted a non-systematic review of materials, including references listed in the WHO guidelines, documents generated by respective governments for their records, and data in the public domain.

Table 1 summarizes the range of functions for home-based records as reported in earlier studies.^3^^–^^13^^,^^20^^–^^43^ We categorized the expected functions into three levels: data recording and storage (level 1); behaviour change communication (level 2); and monitoring and referral (level 3). Both types of home-based record users (i.e. health-care service users and providers) potentially benefit from all three levels of functions of home-based records.

**Table 1:** Characteristics and functions of home-based records for maternal and child health

Characteristics and functions of records	Type of home-based record			
	Maternal health card or handbook	Child vaccination card	Growth chart	Maternal and child health handbook
**Characteristics of home-based records**				
Type of record	Stand-alone, home-based record: specific to reproductive and maternal health programme	Stand-alone, home-based record: specific to expanded programme on immunization	Stand-alone, home-based record: specific to child nutrition programme	Integrated, home-based record: all maternal and child health stages
Document style	One-page card, often foldable; or 20–30 page bound booklet	One-page card, often foldable	One-page card, often foldable	40–60-page bound booklet
Target beneficiary	Pregnant women and mothers	Children	Children	Pregnant women, mothers and children
**Functions of home-based records by level and user**				
Level 1 function: data recording and storage				
For target beneficiary	Makes personal reproductive and maternal data (on pregnancy, childbirth, postpartum and between pregnancies) readily available and accessible at home, and more mobile^5^^,^^22^	Makes personal child immunization data readily available and accessible at home, and more mobile^3^^,^^23^	Makes personal childhood anthropometric data in a line chart readily available and accessible at home, and more mobile^4^	Makes personal data at all stages of maternal and child health (pregnancy, childbirth, postpartum and newborn care; child immunization, growth and development) readily available and accessible at home, and more mobile^6^^–^^13^^,^^20^^,^^21^^,^^24^^–^^34^ Often serves as the documented evidence for eligibility for free or subsidized health-care and other social services, and for assessing performance of results-based financing^30^^,^^35^
For health-care workers	Serves as the reliable source of individuals’ reproductive and maternal data for appropriate clinical decisions^22^ and for health statistics, surveys and research^22^	Serves as the reliable source of individual children’s immunization data for clinical decision-making (e.g. avoiding missed vaccination opportunities)^23^ and for health statistics, surveys and research.^36^ Saves costs related to unnecessary vaccinations through double-checking children’s previous immunization history on the card^23^^,^^37^	Assists health-care workers to detect child malnutrition and intervene in a timely way^4^	Increases clinical efficiency by allowing health-care workers to check records and avoid unnecessary repetition of questions, examinations and vaccinations on maternal and child health.^19^ Serves as the reliable, comprehensive source of individuals’ maternal and child health data for health statistics, surveys and research^36^^,^^38^^,^^39^
Level 2 function: behaviour change communication				
For target beneficiary	Equips pregnant women with knowledge about danger signs and lifestyle changes, to promote self-management during pregnancy, postpartum and between pregnancies.^22^ Often guides pregnant women and family members on upcoming care visit appointments of maternal services^22^	Often guides mothers or other caregivers on the timing of upcoming vaccinations, by enabling them to refer to the child vaccination schedule incorporated in child vaccination cards^23^^,^^40^^,^^41^	Sometimes equips mothers or other caregivers with knowledge about child malnutrition, by enabling them to refer to supplementary text and pictures about appropriate feeding practices (including breastfeeding) in relation to growth charts^42^	Equips and empowers pregnant women, mothers or other caregivers with knowledge on the importance of using health services and of practising home-based care for maternal and child health (during pregnancy, childbirth and postpartum and newborn stages; child immunization, growth monitoring, feeding practices, developmental assessment, and childhood illnesses).^7^^,^^20^ 26,27,35,40 Often triggers positive behaviour changes among family members, to improve and ensure maternal and child health (e.g. family financial preparedness for childbirth and changing husbands’ smoking habits).^7^^,^^8^ Often provides pregnant women, mothers and other caregivers with personalized information on upcoming care visit appointments^9^
For health-care workers	Provides health-care workers with key health education on risks and care during pregnancy, postpartum and between pregnancies^22^	Facilitates communication between mothers and other caregivers and health-care workers on past immunization results and future immunization requirements and planning^23^^,^^40^^,^^41^	NA	Facilitates health-care workers’ guidance for mothers and other caregivers on dos and don’ts during all stages of maternal and child health care^7^^,^^20^^,^^24^^–^^26^^,^^29^
Level 3 function: monitoring and referral				
For target beneficiary	Promotes self-monitoring of maternal health status during pregnancy, postpartum and between pregnancies.^43^ Increases mothers’ self-control over pregnancy.^43^ Empowers pregnant women to recognize risks and to self-refer to a higher or lower level health facility^22^	Promotes self-monitoring of children’s vaccination status by mothers or other caregivers, for better self-planning for upcoming child immunization visits.^23^ Increases mothers’ or other caregivers’ sense of ownership over their children’s health^23^	Provides mothers and other caregivers with opportunities to self-monitor their children’s nutritional status^42^	Promotes overall and continuous self-monitoring of maternal and child health status by mothers and other caregivers.^7^^–^^10^^,^^28^^,^^29^^,^^33^^,^^35^^,^^43^ Increases self-control over maternal and child health that leads to greater satisfaction and self-decision-making.^8^^,^^43^ Empowers mothers to recognize risks and to self-refer to a higher or lower level health facility^8^^,^^20^^,^^33^^,^^43^ Promotes interaction and thereby attachment between mothers or caregivers and their children through self-monitoring child development^7^^,^^27^^,^^33^^,^^35^
For health-care workers	Serves as the means of promoting continuity of care throughout a woman's reproductive life.^22^ Empowers community health-care workers to recognize risks and, if necessary, refer mothers to a higher level health facility.^22^ Serves as the referral form to a higher or lower level health facility along with chronological data of current pregnancy^22^	Serves as the reliable source of child’s individual vaccination data to be shared across different health facilities^23^	NA	Serves to promote a continuum of care throughout all stages of maternal and child health care.^7^^,^^9^^,^^26^^,^^28^^,^^29^^,^^33^^,^^35^^,^^38^^,^^43^ Serves as a personalized checklist that enables health-care workers to adhere to national clinical protocols.^24^ Serves as the referral form to a higher or lower level health facility along with chronological data of maternal and child health^32^^,^^33^^,^^44^

NA: not applicable.

First, data recording and storage (level 1) is, by definition, the minimum common function of all home-based records. Home-based records make personal data related to maternal and child health care readily available and accessible at home. Home-based records can be taken to and used in different facilities, whereas facility-based records are attached to a specific facility. When data are appropriately recorded, the likelihood that home-based records serve as the reliable documented source of individuals’ health data increases.^35^ Those individuals’ health and biological data can be used not only for health care, but also for other relevant purposes. Some home-based records are designed to serve as the birth certificate, and sometimes even as a reference for local civil registration systems, such as in Burundi.^30^ This function helps home-based records serve as the documented evidence for home-based record owner’s eligibility for subsidized health and other social services, such as free maternal and child health services according to child’s age.^30^ Home-based records can be designed to serve as one of the data sources for performance assessments of health-care workers or health facilities, particularly when connected to performance-based financing.^30^ By double-checking children’s immunization histories recorded in home-based records, over-vaccination can be avoided.^37^ Home-based records often function as a reliable source of data for health surveys and research, for example, for estimating essential programme coverage or confirming the reliability of estimates.^36^^,^^38^^,^^39^

Second, behaviour change communication (level 2) is expected in certain types of home-based records, typically in integrated, rather than stand-alone records. Health guidance pages embedded in records help mothers to practise home-based self-learning and peer education for taking necessary action on maternal and child health-related issues. The pages also help health-care workers to deliver educational messages more effectively. The level 2 function of home-based records enables mothers to be equipped with knowledge about danger signs and appropriate lifestyle changes during pregnancy and child care. Mothers can be given guidance on the appropriate timing of services, such as vaccination schedules, and how to practise home-based care.^7^^,^^20^^,^^22^^,^^23^^,^^25^^,^^26^^,^^29^^,^^31^^,^^39^^,^^41^^,^^42^Personalized guidance, such as upcoming service appointments, can be recorded by health-care workers. The function of triggering family members’ positive behaviour changes was reported in several earlier studies on integrated home-based records. For example, fathers may be encouraged to quit smoking, make financial preparations for their baby’s delivery, pay attention to keeping the newborn appropriately warm and increase interaction with their children to benefit early child development.^7^^,^^8^^,^^27^ A key function at level 2 is supporting health-care workers in effectively conveying health messages to mothers and caregivers. For example, the home-based record can provide guidance on the respective stages of maternal and child health care and on seamless use of services across the continuum of care.^22^^,^^23^^,^^25^^,^^29^^,^^40^^,^^41^

Third, monitoring and referral in home-based records (level 3) enables health-care workers to correctly and efficiently track the personal health data and treatment histories of clients. This function is important for maternal and child health services because pregnant women and mothers usually receive health services at different facilities at different stages. For instance, women may visit health centres for antenatal care and child immunization, but go to hospital for delivery.^7^^,^^9^^,^^22^^,^^23^^,^^26^^,^^29^^,^^32^^,^^33^^,^^35^^,^^38^^,^^43^ Clients’ strategic choice of facilities according to their own health-care needs is increasingly common not only in high-income countries but also, more recently, in low- and middle-income countries.^28^^,^^32^ Even those women who originally planned to use maternal and child health services at a single facility can benefit when the home-based record serves as a referral letter to other facilities. Some home-based records require mothers and caregivers to record the results of self-monitoring before and after service appointments. This allows health-care workers to attend to maternal and child health-related issues that are observable only at home, such as child feeding, child development or maternal depression.^8^^,^^27^^,^^35^^,^^45^The level 3 function aims to promote continuous self-monitoring, thereby empowering mothers and caregivers to recognize and address health risks via self-care or self-referral to a higher or lower level of health facility.^8^^,^^20^^,^^32^^,^^33^^,^^43^The process of self-monitoring and self-recording on child development milestones can provide mothers and caregivers with an opportunity to increase interaction with and attachment to their children.^27^^,^^46^

## Function–capacity framework

**Fig. 1:**
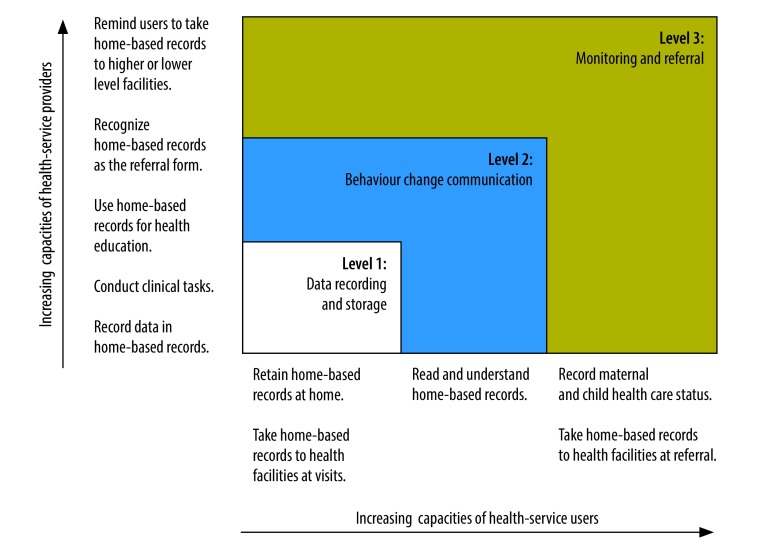
Hypothetical progression of users’ capacities that enable home-based records to function as designed

Categorizing the various functions of home-based records raises the question whether and to what extent the users are knowledgeable and skilful enough to make the expected functions happen. More importantly, what capacities do users need to realize these functions? This question has rarely been raised, despite its importance as a critical determinant of the type of home-based record to be employed. We assume that different levels of home-based record functions require users to be equipped with different types and levels of capacities. Fig. 1 illustrates the hypothetical capacity requirements of users of home-based records in relation to the three levels of function that we have outlined. Note that users’ capacities here encompass not only knowledge and potential abilities, but also motivation and attitudes that enable practices.

First, to keep a home-based record functioning as a data recording and storage tool, users of the records need only minimum capacities. Health-care workers need to be capable of recording the results of health-care services in the records. Mothers and caregivers need to be capable of retaining the records at home without damaging, misplacing or losing them and to take them to the points of services. The contents of home-based records need not be fully understood by mothers and caregivers at this level of function. However, it is not clear to what extent home-based records are functioning as data recording and storing tools in low-literacy settings. WHO has indicated that poor retention of home-based records by mothers and caregivers needs to be addressed, while acknowledging that some countries have achieved success in home-based record retention.^1^ Loss of home-based records was reported as the critical issue by several earlier studies too.^2^^,^^3^ A low level of literacy among mothers is one factor that could affect receipt and loss of home-based records by mothers, although factors unconnected to users capacities, such as poorly functioning health systems, could also affect the use of these records.^1^

Second, for home-based records to function as a behaviour change and communication tool, mothers and caregivers need greater capacities beyond literacy. To enable a home-based record to trigger behaviour change through self-learning and peer-education, mothers and caregivers should be able not simply to read and understand the contents of guidance pages, but also to incorporate them into practice, when needed, with the help of other family members. Health-care workers should not simply be literate, but also technically skilled enough to translate the information into the local maternal and child health context. Therefore, health-care workers need to provide guidance to women about the content and value of the home-based record.

Third, if home-based records are to function as a monitoring and referral tool, health-care workers need to be equipped with the knowledge and skills to use the records for appropriate, comprehensive clinical decisions. This needs to happen at all levels of health care (primary, secondary and tertiary) within maternal and child health programmes and in both the public and private sectors. Mothers and caregivers need to present the record at different facilities to allow health-care workers to refer to and update the data and hence leverage the inter-facility mobility of home-based records. To record data on child development, mothers and caregivers need the capacity in their daily routine to objectively observe their children’s behavioural and cognitive responses against the child development milestones in home-based records.^45^

## Filling the capacity gap

Fig. 1 assists health policy-makers in identifying the discrepancy between the functions a health system demands and the functions that can be realized within the capacities of current users. When a home-based record designed for a lower-level function is employed in a setting with users of higher capacities, health policy-makers can, in theory, expand the functions to cover the higher levels. On the other hand, when a record designed for higher-level functions is employed in settings with users of lower capacities, policy-makers need to downgrade functions or add supplementary elements to the record and its operation. Typical supplementary elements include designing records for greater user-friendliness and adding supportive interventions for increasing the capacities of record users.

Accurate understanding of users’ current capacities will inform a more user-friendly and effective design. Some health-care workers are not motivated to record clinical data in home-based records because they see the task as an additional workload.^3^ To encourage recording, the layout of entry columns in a home-based record should be carefully designed to enable selected clinical data to be efficiently and correctly transcribed from facility-based records to, for example, the maternal and child health logbook or child immunization logbook.^47^ Losing and not receiving home-based records are concerns.^2^ Reports from Indonesia, Kenya, Uganda, and West Bank and Gaza Strip suggest that records with graphics and illustrations were more understandable and attractive to illiterate or less literate mothers and might have contributed to higher coverage and lower loss of home-based records.^44^ More user-friendly design is effective, particularly when the health system requires level 2 functions, but users’ capacities remain at the level 1 function. Some home-based records include vouchers for immunization services or food rations as in Japan.^10^^,^^35^ Embedding incentive vouchers in records often increases a sense of ownership and hence retention of home-based records by mothers and caregivers. This approach is effective when users may not have the capacity to fulfil level 1 functions. Minimizing the use of medical terms and avoiding wordiness in the texts increases the feasibility of designing home-based records for higher-level functions.^12^^,^^44^

Supportive interventions for increasing users’ capacities and orientation and refresher training for health-care workers are essential for realizing all levels of home-based record functions.^1^ The contents of training for health-care workers could be designed to fill the gaps between current capacities and required capacities. For instance, in-service training on health education methods are an effective means of increasing the feasibility of level 2 functions in home-based records.^48^^,^^49^ Training during pre-service education could be an efficient strategy that pre-empts a need for filling gaps in health-care workers’ capacities. In some countries, health professional associations award specialist accreditation to those who have practised quality care including health education by using home-based records. Training programmes conducted by nongovernmental organizations could help ensure nationally standardized home-based records are used in both public and private health facilities.^15^^,^^28^ Some countries found that follow-up supportive supervision reinforces the capacities of health-care workers.^46^ Particularly when training programmes are not readily available and accessible for health-care workers; guides or manuals serve as practical tools that assist health-care workers towards smoother and more efficient use of records.^34^^,^^50^ Health-care workers’ guides are often developed at the time of development or revision of home-based records.^33^

## Conclusion

To accommodate the growing demands of national health policies and health systems, policy-makers tend to make overambitious plans for home-based records. As a result, important functions of these records often remain underused, leading to loss of confidence in the records and to a waste of resources and opportunities for care.

There are several key capacities of home-based record users that act as determinants of optimal use of home-based records. At a minimum level, mothers and caregivers should be capable of carefully retaining home-based records and taking them to the points of service. For higher levels of functions, mothers need the capacities to translate maternal and child health-related messages into their own context and take sensible decisions about actions over time. Health-care workers need the basic capacity to undertake stock management of records and to record data in records. At higher levels of function, they need the capacity to explain the data and health-related messages to mothers and caregivers and to translate these for appropriate clinical decisions. When designing a home-based record, the capacities of both types of users must be assessed and considered for optimizing the use of the records.

Today, several international guides and manuals on home-based records have been published by WHO.^1^^,^^3^^,^^5^ However, these guides do not fully address ways of strategically selecting the most suitable home-based record for each country, in accordance with the capacities that users currently have or potentially would have. One reason for this may be an absence of evidence that identifies the relationship between the level of home-based record functions and users’ capacities. Mismatches between the expected functions of records and the realities of users’ capacities need to be examined for the optimal use of home-based records in maternal, newborn and child health care.
